# Mountains, Lakes, and Ancient Drainage Networks Sculpt the Phylogeographic Architecture of the Stream Headwater Fish *Acrossocheilus kreyenbergii* in China

**DOI:** 10.3390/genes16121393

**Published:** 2025-11-21

**Authors:** Yun Chen, Guangmin Deng, Ziyu Le, Cuizhang Fu

**Affiliations:** State Key Laboratory of Wetland Conservation and Restoration, National Observations and Research Station for Wetland Ecosystems of the Yangtze Estuary, Ministry of Education Key Laboratory for Biodiversity Science and Ecological Engineering and Institute of Eco-Chongming, School of Life Sciences, Fudan University, Shanghai 200438, China; 20110700132@fudan.edu.cn (Y.C.); 21110700097@m.fudan.edu.cn (G.D.); 23210700105@m.fudan.edu.cn (Z.L.)

**Keywords:** Cyprinidae, genetic diversity, phylogeography, Yangtze River, Zhe–Min Uplift, stream capture

## Abstract

Background: Phylogeographic surveys of obligate freshwater fishes could serve as a pivotal lens through which the biological footprints of historic drainage rearrangements can be deciphered. Methods: Focusing on the headwater-restricted cyprinid *Acrossocheilus kreyenbergii* in the Pearl, Yangtze, and Huai river basins, we examined variations in mitochondrial cytochrome *b* gene (*Cyt b*) to elucidate the phylogeographic architecture and evolutionary history of this stream fish in South–Central China through integrative analyses of phylogeny, ancestral area reconstruction, genetic structure, and population demography. Results: A time-calibrated phylogeny recovered two primary lineages, K-I and K-II, which diverged ca. 2.15 Ma: K-I split into K-Ia (Huai River) and K-Ib (Yangtze–Poyang Lake catchment) at 1.53 Ma, whereas K-II gave rise to K-IIa, K-IIb, and K-IIc through sequential divergences at 1.29 Ma and 0.83 Ma, with K-IIa restricted to the Poyang Lake catchment. K-IIb was shared between the Poyang Lake catchment and the Qiupu River (Yangtze basin), and K-IIc was distributed in the Xijiang River (Pearl basin) as well as the Yangtze–Dongting Lake catchment. Conclusions: Our findings reveal that the phylogeographic architecture of *A. kreyenbergii* was sculpted by a succession of geologic and anthropocentric events: the Late-Cenozoic collapse of the Zhe–Min Uplift first fractured its range; the intervening Mufu–Lianyun–Luoxiao Mountains then acted as a persistent barrier; the large waters of Poyang and Dongting Lakes served as biological filters; and the 2200-year-old Lingqu Canal—constructed during the Qin dynasty—briefly re-established a corridor for gene flow. Together, these forces disrupted and reorganized the species’ genetic connections, leaving a visible imprint today.

## 1. Introduction

Phylogeographic research on freshwater fishes continues to play a pivotal role in uncovering the underlying mechanisms that shape genetic variations within freshwater fish biodiversity across isolated drainages and river networks. Phylogeography of obligate freshwater fishes has become a cornerstone for deciphering the biological signatures left by drainage rearrangements because these fishes can disperse only through historically and currently connected river courses [1,2,3]. Such rearrangements appear in two dominant forms: stream capture and coastal paleo-drainage connections [4,5,6,7]. Stream capture occurs when tectonic uplift or headward erosion severs a tributary from one river and diverts it into an adjacent basin [8,9]. Coastal paleo-drainage connections, by contrast, arise when falling Pleistocene sea levels force separate coastal rivers to coalesce downstream, fusing their catchments into a single transient drainage network; those rivers subsequently became separated due to rising sea levels as the Pleistocene ended [10,11]. Stream capture and coastal paleo-drainage connections have become the interpretive cornerstones of piscine phylogeography, routinely invoked to explain both abrupt genetic breaks and the paradoxical lack of structure among freshwater fish now isolated in separate coastal basins [12,13,14,15,16,17,18,19,20,21,22,23]. However, an expanding body of evidence shows that geographical features, Pleistocene climatic oscillations, and the differential erosion of contrasting rock types are powerful engines of lineage divergence in freshwater fish [24,25,26,27,28,29,30,31,32,33,34,35,36]. In South–Central China, recent studies have revealed the critical role of the Zhe–Min Uplift, Poyang Lake, and the Lingqu Canal in shaping phylogeographic patterns of stream fishes [18,33].

**Figure 1:**
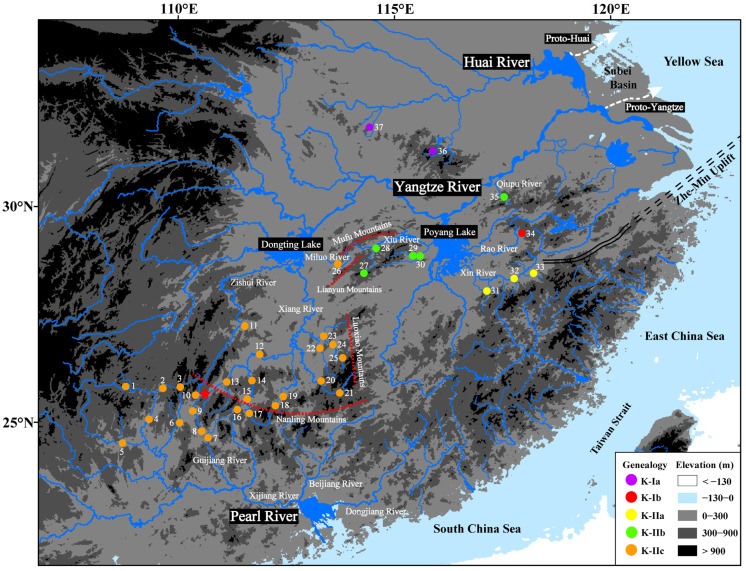
Geographic distributions of the 37 collection sites and five *Cyt b* sub-lineages recovered in *A. kreyenbergii*. White dashed lines delineate the Proto-Yangtze River and the Proto-Huai River, after [47,51], respectively. Major drainage divides are shown in red dashed lines; the Zhe–Min Uplift is indicated by black dashed lines [41]. The Lingqu Canal—an artificial waterway excavated during the Qin Dynasty—is marked by a red star.

The Zhe–Min Uplift—also known as the Fukien–Reinan Massif or the Zhejiang–Fujian Uplift—is situated at the junction of the Yellow Sea and the East China Sea [37,38]. It is a composite orogenic belt trending northeast–southwest, uplifted during the middle to late Mesozoic, stretching from the Southern Korean Peninsula to the Northern East China Sea margin [39,40]. This belt forms a high-relief barrier between the continental interior and the proto-Pacific coast, effectively preventing the ingress of seawater into the paleo-Yellow Sea region [41]. Several minor marine incursions interrupted the paleo-Yellow Sea region between approximately 1.66 and 1.0 million years ago (Ma) during the late Early Pleistocene, a consequence of the initial subsidence of the Zhe–Min Uplift [41,42,43,44]. This subsidence culminated in a major transgression around 0.8 Ma, which established normal marine environments in the Yellow Sea due to the ongoing tectonic subsidence of the Zhe–Min Uplift [44,45,46,47]. Prior to the subsidence of the Zhe–Min Uplift (Figure 1), the rivers of the proto-Yangtze and proto-Huai systems traversed the Subei basin and discharged into the paleo-Yellow Sea region, implying that the two systems may have once been linked [48,49,50,51]. A major river diversion redirected the Yangtze River’s flow southward into the East China Sea between approximately 0.8 Ma and 0.6 Ma, culminating in its modern configuration [47,52]. Thus, we propose that the Zhe–Min Uplift formerly served as a dispersal corridor for fish between the Yangtze and Huai rivers, whereas its subsequent subsidence led to their isolation and subsequent genetic divergence.

Poyang and Dongting Lakes (Figure 1)—two immense water bodies in the middle Yangtze basin—each cover mean areas of ~3210 km^2^ and ~1148 km^2^, respectively [53]. The Poyang Lake catchment is anchored by the lake and its five major tributaries—the Xiu, Rao, Xin, Fu, and Gan Rivers—whereas the Dongting Lake system comprises the lake and the Miluo, Xiang, Zi, Yuan, and Li Rivers. The Poyang Lake and Dongting Lake basins originated in the Late Mesozoic and experienced regional-scale subsidence during the Early–Middle Pleistocene [54,55,56,57,58,59,60]. The Mufu, Lianyun, and Luoxiao mountain ranges collectively form the drainage divide, separating the Poyang and Dongting Lake sub-catchments. Earlier work has documented striking contrasts in stream fish assemblages on either flank of these ranges, indicating that the mountains act as a major barrier to fish dispersal [61,62]. We suggest that this topographic isolation has produced detectable genetic divergence between fish populations in the Poyang and Dongting sub-catchments.

Monaghan et al. [63] first reported that the alpine stream insect mayfly *Baetis alpinus* exhibits pronounced genetic divergence between populations immediately above and below natural lakes within the river networks, suggesting that lentic stretches act as strong barriers to dispersal. Pelicice et al. [64] extended this insight, demonstrating that the lotic-to-lentic shift imposed by reservoirs can act as an ecological filter, severely limiting the downstream movement of rheophilic fish. The same “lake-barrier” signature now emerges from the Poyang Lake catchment: Yang et al. [28] and Li et al. [18] uncovered deep phylogeographic breaks between tributary populations of two small rheophilic fish species—the gudgeons *Huigobio chenhsienensis* and *Sarcocheilichthys parvus*—demonstrating that standing water repeatedly disrupts gene flow, even within a vast flood-pulse wetland. This leads us to consider that both Poyang and Dongting Lakes function as hard barriers to gene flow among their respective tributary populations of rheophilic fishes.

The Nanling Mountains (Figure 1) serve as the drainage divide between the Pearl River and Yangtze River basins. Although the Nanling divide has been reported to coincide with phylogeographic breaks between Pearl- and Yangtze-basin fishes [18,65,66], an equally large body of data found no appreciable genetic structure across the same boundary [29,67,68,69,70,71,72]. The Lingqu Canal (Figure 1), constructed during the Chinese Qin Dynasty over 2200 years ago, was designed to link the headwaters of the Xiang River (a major tributary of the Dongting Lake catchment) with the Gui River (a principal tributary of the Xijiang catchment in the Pearl River basin) [73]. This hydrological connection enables the flow of water from the Xiang River into the Gui River across the drainage divide [74], potentially opening a dispersal corridor through which their respective fish species can expand their ranges. A few phylogeographic studies on freshwater fishes have already implicated the Lingqu Canal as a pivotal conduit for population exchange between the Xijiang and Dongting Lake catchments [67,68,69,70]. Accordingly, we propose two alternative—and testable—scenarios: (1) the Nanling range functions as a long-term barrier driving vicariant divergence between Pearl- and Yangtze-basin fish lineages, and (2) the Lingqu Canal has repeatedly acted as a dispersal corridor, enabling the uni- or bidirectional colonization of headwater fishes between the Xiang (Yangtze) and Gui (Pearl) Rivers.

*A. kreyenbergii* (Cypriniformes: Cyprinidae) is an upland small-stream headwater specialist, rarely exceeding 16 cm in standard length, and is endemic to China [75]. This fish species is distributed across three major river systems (Figure 1): the Pearl River, Yangtze River, and Huai River basins [75,76,77,78]. Within the Pearl River basin, it is found exclusively in the Xijiang River. In the Yangtze River basin, its range includes the rivers of Xiang, Zi, and Miluo rivers within the Dongting Lake catchment, the Xiu, Rao, and Xin rivers within the Poyang Lake catchment, as well as the Qiupu River. In the Huai River basin, it is restricted to headwater streams of the upper reaches. *A. kreyenbergii* typically inhabits the middle and upper reaches of pristine headwater streams, showing a clear preference for substrates of sand, pebble, gravel, or rock [79]. Their diet primarily consists of benthic algae, with aquatic insects also being consumed. *A. kreyenbergii* attain sexual maturity at the age of 2 years, with a breeding season that extends from late spring through early autumn. During this period, they lay eggs that adhere to rocks or other hard substrates [79].

Using mitochondrial cytochrome *b* gene (*Cyt b*) sequences and a suite of phylogeographic analyses, we determined the evolutionary history of the Chinese endemic rheophilic cyprinid *A. kreyenbergii*. This study offers a detailed example of how landscape evolution governs intraspecific genetic structure in East-Asian freshwater fishes and provides a comparative reference for other cyprinids. We evaluate four non-exclusive hypotheses that could explain the lineage divergence within the species: (1) The Late-Cenozoic collapse of the Zhe–Min Uplift reorganized drainage patterns and led to a vicariant split between the Yangtze and Huai drainage populations. (2) The Mufu–Lianyun–Luoxiao mountain chain isolates the Poyang and Dongting tributaries, producing distinct genetic structures between these sub-catchments. (3) Poyang and Dongting Lakes act as inland barriers that restrict gene flow among their respective tributary assemblages. (4) Either the Nanling Range has long functioned as a vicariant barrier separating the Pearl- and Yangtze-basin lineages, or the Lingqu Canal has repeatedly served as a dispersal corridor enabling uni- or bidirectional headwater exchange between the Xiang (Yangtze) and Gui (Pearl) Rivers.

## 2. Materials and Methods

### 2.1. Specimen Collection

From March 2012 to August 2024, with the assistance of local fishermen using gill nets, we collected 223 *A. kreyenbergii* specimens from 36 sites across the Pearl, Yangtze, and Huai River basins, encompassing the species’ entire known range (Figure 1; Table S1). Fish collections received approval from the Animal Ethics Committee of Fudan University and were conducted in compliance with the Chinese national standard “Laboratory Animals—Guideline for Ethical Review of Animal Welfare (GB/T 35892-2018 [80])”. The field-caught fish were anesthetized using a 0.25 mL/L aqueous solution of Eugenol (Shanghai Acmec Biochemical Technology Co., Ltd., Shanghai, China) until they became unconscious. After euthanasia, the fish were fixed in 75% ethanol (Sinopharm Chemical Reagent Co., Ltd., Shanghai, China) for field preservation, transferred to 95% ethanol (Sinopharm Chemical Reagent Co., Ltd., Shanghai, China) for long-term storage, and deposited in the Zoological Museum of Fudan University (see Table S2 for collection numbers). An additional mitochondrial *Cyt b* sequence of *A. kreyenbergii* from the Huai River basin was retrieved from GenBank (accession number KJ817184) [77].

### 2.2. DNA Sequencing and Alignment

High-salt extraction was used to isolate genomic DNA from dorsal-muscle tissue [81]. Novel PCR primers GluF-Acr (5′-GAGACCAATGACTTGAAGAAC-3′) and ProR-Acr (5′-GTTTAGTTTAGAATTCTGGCTTTGGG-3′) were used to amplify the complete *Cyt b* gene. Each 50 μL PCR reaction mix contained 25 μL of 2 × premix (Nanjing Vazyme Biotech Co., Ltd., Nanjing, China), 1 μL of each primer (Sangon Biotech (Shanghai) Co., Ltd., Shanghai, China), 1 μL of genomic DNA, and 22 μL of ddH_2_O (Sangon Biotech (Shanghai) Co., Ltd., Shanghai, China); amplifications were run at 95 °C for 3 min, 35× (95 °C for 15 s, 51.8 °C for 15 s, 72 °C for 60 s), and 72 °C for 5 min; and subsequently sequenced (Sanger) at Shanghai JieLi Biotech, China. Raw reads were trimmed and assembled in Sequencher v5.4.6 (Gene Codes, Ann Arbor, MI, USA) with manual proof-reading, aligned using MAFFT v7.526 [82] under FFT-NS-2, and translated using DAMBE v7.3.32 [83] to confirm uninterrupted coding frames; *Cyt b* haplotypes were defined using DnaSP v6.12.01 [84]. The molecular features of the *Cyt b* sequences were analyzed using MEGA v11.0.13 [85].

### 2.3. Phylogeny and Divergence-Time Estimation

A time-calibrated *Cyt b* haplotype phylogeny of *A. kreyenbergii* was inferred using BEAST v2.7.3. [86]. Because no fossils are available to calibrate the genus *Acrossocheilus*, divergence times were estimated with a cyprinid-specific *Cyt b* mutation rate of 1.045% per site per million years derived from dated Asian *Carassius* fossils [24]. The best-fit nucleotide substitution model TN93 for the *Cyt b* alignment was automatically selected using bModelTest v1.3.3 [87]. The tree prior was set to the birth–death model. A likelihood-ratio test in DAMBE v7.3.32 [83] failed to reject a global molecular clock for the *Cyt b* data (χ^2^ = 70.481, *d.f.* = 64, *p* = 0.270); so, analyses were run under a strict clock assumption. Two independent MCMC runs of 400 million generations were sampled every 20,000 steps. The convergence was determined based on ESS > 200 for each parameter in Tracer v1.7.1 [88]. After discarding 30% burn-in, trees and logs were merged with LogCombiner, summarized with TreeAnnotator, and displayed in FigTree v1.4.4 [89]. Based on the phylogeny of *Acrossocheilus* [90], *Acrossocheilus wenchowensis* and *Acrossocheilus fasciatus* were designated as outgroups, with GenBank accession numbers PX094885–PX094888 obtained in this study.

To visualize intraspecific variation, a median-joining network was built from the *Cyt b* haplotypes in Network v10.2.0 [91] under the minimum-mutation criterion and then color-coded according to the river basin.

### 2.4. Genetic Structure and Population History

The observed genetic diversity was quantified as haplotype (*h*) and nucleotide (*π*) diversity using DnaSP v6.12.01 [84]. The total and pairwise Φ_ST_ values among the Pearl River, Dongting Lake catchment, Poyang Lake catchment, Qiupu River, and Huai River systems were computed using Arlequin v3.5.2.2 [92]. To assess the phylogeographic structure, Nei’s G_ST_ and N_ST_ were calculated using PermutCpSSR v2.0 [93]. G_ST_ compares haplotype frequencies without considering the genetic distance, whereas N_ST_ incorporates the number of mutational steps between haplotypes. A permutation test (1000 replicates) was used to determine whether N_ST_ was greater than G_ST_; rejection of the null hypothesis (G_ST_ = N_ST_) indicates that genealogically similar haplotypes are geographically clustered, consistent with isolation-by-distance or historical fragmentation. SAMOVA v2.0 [94] was employed to perform spatial analysis of molecular variance (SAMOVA) and to identify the population configuration that maximized the Φ_CT_ statistic. The SAMOVA partition biologically represents the optimal genetic clustering of populations that explains the maximum among-group variance, providing a spatially explicit hypothesis about population subdivision and gene flow barriers in a study system.

The demographic history was reconstructed in two complementary ways. First, Arlequin v3.5.2.2 [92] was used to calculate Tajima’s *D* and Fu’s *Fs* neutrality statistics and to fit a sudden expansion model to the mismatch distribution of pairwise differences; a total of 5000 parametric bootstraps provided confidence intervals for the expansion parameters τ, θ_0_, and θ_1_. Second, a Bayesian skyline plot (BSP) was generated in BEAST v2.7.3 [86] under a strict molecular clock, with the *Cyt b* substitution rate set to 1.045% site^−1^ Myr^−1^ [24] and a generation time of 2 years [79]. Two independent MCMC chains were run for 100 million generations, sampling every 5000 steps, and the resulting log files were combined in LogCombiner after discarding 20% as burn-in; the BSP was visualized in Tracer v1.7.1 [88].

### 2.5. Ancestral Area Reconstruction

We employed the R package BioGeoBEARS v0.2.1 [95] to test competing hypotheses of historical biogeography and to infer ancestral ranges. The time-calibrated *Cyt b* haplotype tree served as the input topology. Five biogeographic units were delimited: the Pearl River, the Dongting Lake catchment, the Poyang Lake catchment, the Qiupu River, and the Huai River. Each biogeographic unit was treated as a discrete area, and haplotypes were assigned to the associated units from which they were sampled (Table S1). Six models were compared: DEC, DIVALIKE, and BAYAREALIKE, together with their “+J” variants that allow the founder event speciation (DEC + J, DIVALIKE + J, BAYAREALIKE + J). The model ranking relied on the second-order Akaike information criterion corrected for small sample size (AICc) and its associated weight (AICc_wt), which balances the likelihood against the parameter number. Ancestral ranges were then reconstructed under the best-supported model.

## 3. Results

### 3.1. Phylogeny, Divergence Time, and Geographic Distribution

A total of 224 *Cyt b* sequences (1141 bp) of *A. kreyenbergii*, obtained from 37 sampling localities across the three river basins (Figure 1; Table S1), defined 62 haplotypes (GenBank accession numbers: KJ817184 [77], PV699891–PV699950, PX094884). These sequences contained 125 variable sites, 98 parsimony-informative sites, and a GC content of 44.5%.

**Figure 2:**
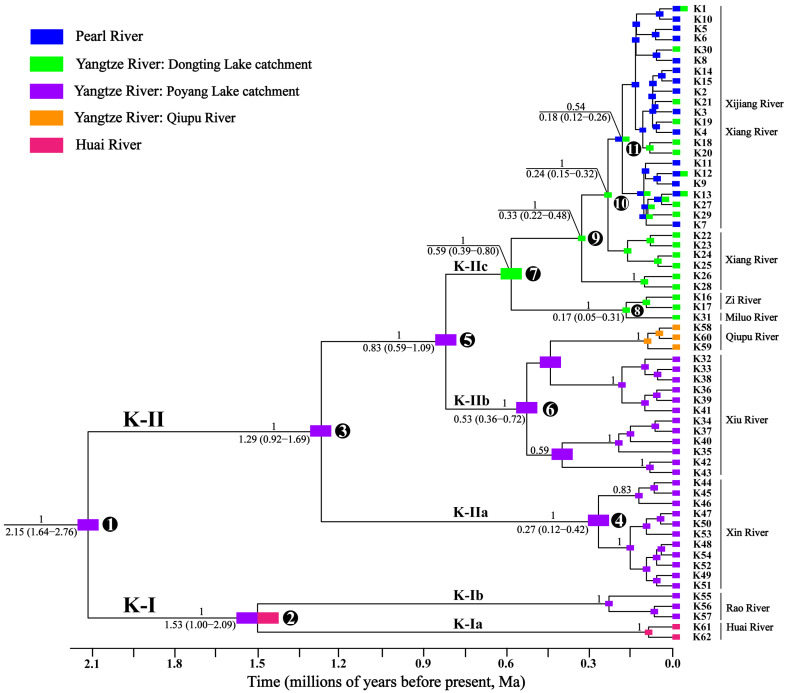
Ancestral-area reconstruction and time-calibrated Bayesian phylogeny of *A. kreyenbergii* inferred from 62 *Cyt b* haplotypes. Posterior probabilities (>0.5) are shown above branches; mean divergence times (95% HPD) are shown below; and key nodes are marked by black-circled numbers.

The results of the time-calibrated phylogeny reconstruction (Figure 2) identified two major lineages (K-I and K-II) that diverged at approximately 2.15 Ma. Lineage K-I subsequently split into two sub-lineages (K-Ia and K-Ib) around 1.53 Ma. Meanwhile, Lineage K-II diverged into three sub-lineages (K-IIa, K-IIb, and K-IIc) at approximately 1.29 Ma and 0.83 Ma, respectively. The spatial distribution of lineages and sub-lineages exhibited conspicuous geographic structure (Figure 1 and Figure 2).

**Figure 3:**
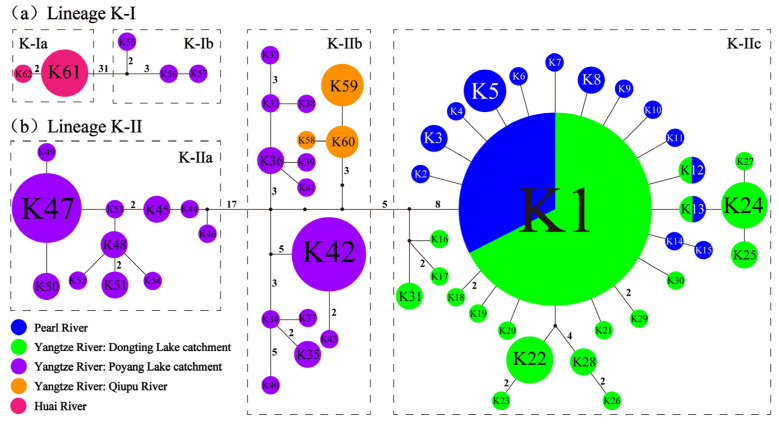
Median-joining networks depicting the genealogical relationships among *Cyt b* haplotypes of *A. kreyenbergii*: (**a**) Lineage K-I and (**b**) lineage K-II. Haplotypes are coded as numbered circles whose sizes scale with their observed frequencies; black nodes denote missing or unsampled intermediate haplotypes. The number of mutational steps separating haplotypes more than single step is given along each branch.

Figure 3 illustrates the haplotype networks. The color code matches that of Figure 2. The two major lineages, K-I and K-II, are separated by 37 mutational steps. Within lineage K-I, sub-lineages K-Ia and K-Ib are connected by 31 mutation steps (Figure 3a). K-Ia is distributed in the Huai River, whereas K-Ib is found in the Rao River of the Poyang Lake catchment (Yangtze River) (Figure 1 and Figure 2). Within lineage K-II, there are 17 mutational steps between sub-lineages K-IIa and K-IIb and 5 mutational steps between K-IIb and K-IIc (Figure 3b). K-IIa is found in the Xin River of the Poyang Lake catchment (Figure 1 and Figure 2). K-IIb is distributed in the Xiu River of the Poyang Lake catchment and the Qiupu River (Yangtze River) (Figure 1 and Figure 2). K-IIc is found in the Xijiang River of the Pearl River basin, as well as in the Xiang, Zishui, and Miluo Rivers of the Dongting Lake catchment (Figure 1 and Figure 2). Within K-IIc, haplotypes K1, K12, and K13 are jointly present in the Xijiang (Pearl basin) and the Xiang Rivers (Yangtze basin) (Figure 2 and Figure 3).

### 3.2. Ancestral-Area Reconstruction

Selected by the Corrected Akaike Information Criterion as the best-fit model (Table S3), the DEC + J ancestral-area reconstruction traces five pivotal history events (Figure 2): (1) origin of *A. kreyenbergii* in the Poyang Lake catchment of the Yangtze River basin (node 1); (2) dispersal from the Poyang Lake catchment to the Huai River between nodes 1 and 2, followed by vicariance at node 2; (3) dispersal from the Poyang Lake catchment to the Dongting Lake catchment between nodes 5 and 7; (4) dispersal from the Poyang Lake catchment to the Qiupu River between node 6 and its tip; (5) dispersal from the Dongting Lake catchment to the Xijiang River (Pearl River basin) between nodes 10 and 11.

### 3.3. Genetic Diversity and Genetic Structure

For the complete *A. kreyenbergii* dataset, the haplotype diversity (*h*) was 0.7952, and the nucleotide diversity (*π*) was 0.014 (Table 1). Across river basins, the haplotype diversity declined from 0.927 in the Yangtze to 0.286 in the Huai, while the nucleotide diversity peaked at 0.0160 in the Yangtze and dropped to 0.0004 in the Pearl River (Table 1). Within the Yangtze basin, the haplotype diversity ranged from 0.639 in the Qiupu River to 0.886 in the Poyang Lake catchment, and the nucleotide diversity ranged from 0.0007 to 0.0159, respectively (Table 1).

The total Φ_ST_ was 0.4789 (*p* < 0.0001), and the pairwise Φ_ST_ ranged from 0.1182 (Pearl River vs. Dongting Lake catchment) to 0.9911 (Pearl River vs. Huai River) (Table 2). G_ST_ and N_ST_ were 0.390 and 0.852, respectively. In the SAMOVA, the among-group genetic variance (Φ_CT_) plateaued at 0.7672 when K = 4. The optimal partition at this K separated the samples into four units: (1) Pearl River plus Dongting Lake catchment, (2) Poyang Lake catchment, (3) Qiupu River, and (4) Huai River.

### 3.4. Population History

Owing to the small sample size, the demographic history of lineage K-I was not inferred; lineage K-II, in contrast, shows a pronounced phylogeographic structure (Figure 2 and Figure 3), and its three sub-lineages (K-IIa, K-IIb, K-IIc) were therefore analyzed separately.

**Figure 4:**
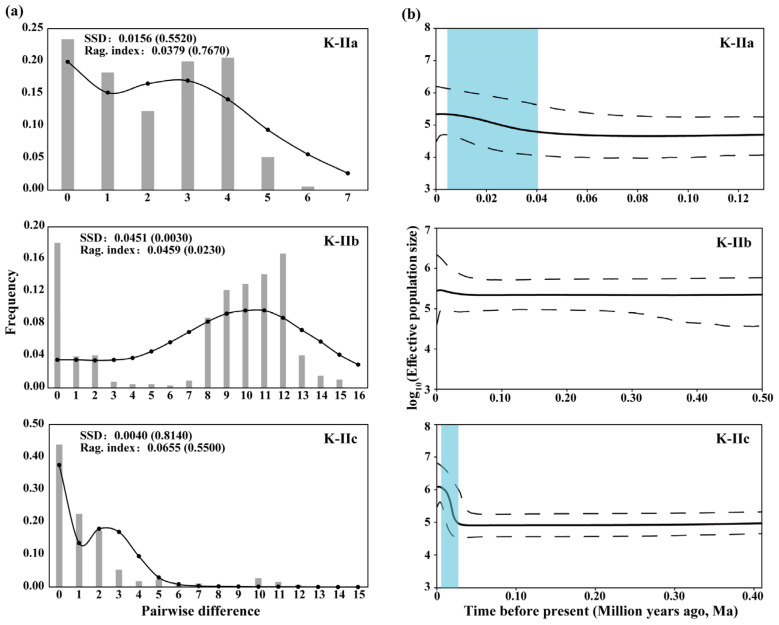
Demographic trajectories of the three sub-lineages in *A. kreyenbergii*. (**a**) Mismatch distributions: observed frequencies are grey bars; the fitted sudden-expansion model is overlaid as a black dotted line. (**b**) Bayesian skyline plot: the solid black curve traces the median log_10_ of effective population size (*Ne*), the grey zone (bounded by black dashed lines) marks the 95% highest posterior density interval, and light-blue shading highlights phases of population growth.

For sub-lineage K-IIa, Tajima’s *D* was negative but non-significant (*D* = −0.5722, *p* = 0.3190), suggesting only a weak excess of rare alleles. Fu’s Fs, however, was strongly negative and significant (*Fs* = −4.3005, *p* = 0.0070), indicating a clear signal of recent population expansion. Although the mismatch distribution is bimodal (Figure 4a), sub-lineage K-IIa fits the sudden-expansion model closely: the sum-of-squared deviations (SSD) = 0.0156 (*p* = 0.5520), and Harpending’s raggedness index (HRI) = 0.0379 (*p* = 0.7670). The Bayesian skyline plot dates the onset of rapid growth at ~0.014 Ma (Figure 4b).

Sub-lineage K-IIb yielded a positive but non-significant Tajima’s *D* (*D* = 0.1734, *p* = 0.6060), hinting at a mild excess of intermediate-frequency variants, whereas Fu’s Fs was weakly negative and likewise non-significant (*Fs* = −0.2935, *p* = 0.4810), providing no clear signal of expansion or contraction. Although the mismatch distribution is unimodal (Figure 4a), the sudden-expansion model is rejected by both SSD = 0.0451 (*p* = 0.0030) and HRI = 0.0459 (*p* = 0.0230). The Bayesian skyline plot nevertheless suggests modest population growth since 0.005 Ma (Figure 4b).

For sub-lineage K-IIc, both neutrality tests were strongly negative and highly significant (Tajima’s *D* = −0.0850, *p* < 0.0001; Fu’s *Fs* = −27.7220, *p* < 0.0001), jointly pointing to a pronounced excess of rare haplotypes consistent with a recent and rapid population expansion. The mismatch distribution exhibited a clear bimodal shape (Figure 4a); nevertheless, it did not deviate from the sudden-expansion model (SSD = 0.0040, *p* = 0.8140; HRI = 0.0655, *p* = 0.5500). The Bayesian skyline plot indicates rapid growth since 0.0097 Ma (Figure 4b).

## 4. Discussion

### 4.1. Main Causes of Lineage Splitting

Our analyses reveal that *A. kreyenbergii* inhabiting the Pearl, Yangtze, and Huai basins lacks basin-specific monophyletic structure. Instead, the species conforms to Avise’s Type-II phylogeographic model [96], in which two deeply divergent lineages (K-I and K-II) co-occur within the Yangtze–Poyang Lake catchment but are confined to separate tributaries (Figure 1 and Figure 2). Ancestral-area reconstruction and molecular dating trace the origin of *A. kreyenbergii* to the Poyang Lake catchment at the onset of the Early Pleistocene at approximately 2.15 Ma (Figure 2). Although the Poyang depression first formed in the Late Mesozoic, only the pronounced Pleistocene subsidence—by re-routing drainages and increasing the area of the depression—generated a lake that matched the area of its modern counterpart [59,97,98]. This vast water body functioned as an impassable biogeographic filter, severing the gene flow among tributaries and leading to the primary divergence of *A. kreyenbergii* into lineages K-I and K-II. Superimposed upon this physical fragmentation, the simultaneous weakening of the East-Asian summer monsoon—together with the attendant cooling and aridification [99,100] —deteriorated freshwater habitats and squeezed populations through severe bottlenecks. The resultant crash in effective population sizes accelerated the stochastic extinction of many intermediate haplotypes, thereby sculpting the deep sympatric split that underpins the Type-II phylogeographic architecture now emblematic of *A. kreyenbergii*.

Within lineage K-I, sub-lineage K-Ia occupies the Yangtze–Poyang Lake catchment, whereas K-Ib is restricted to the Huai River basin. Ancestral-area reconstruction and coalescent analyses indicate that *A. kreyenbergii* colonized the Huai River via downstream dispersal from the Poyang Lake catchment before the late Early Pleistocene; subsequent vicariance split sub-lineages K-Ia and K-Ib around 1.53 Ma (Figure 1 and Figure 2). Divergence between the two sub-lineages is dated to the late Early Pleistocene, in temporal lock-step with the final subsidence of the Zhe–Min Uplift [41,42,43,44]. This supports our hypothesis that fish populations once dispersed between the Yangtze and Huai drainages due to the Zhe–Min Uplift, whereas its subsequent subsidence and marine incursions severed the connection, driving the genetic split. A recent study reported the phylogeographic break in the stream fish *S. parvus* (Cypriniformes: Gobionidae) [18], implicating the Zhe–Min Uplift as a major driver of genetic divergence and speciation across aquatic taxa of the coastal rivers in China.

Within lineage K-II, sub-lineages K-IIa and K-IIb exhibit pronounced phylogeographic structures, in which K-IIa is restricted to the Xinjiang River and K-IIb to the Xiushui River, both tributaries of Poyang Lake. Sub-lineage K-IIc, sampled from the Xiang, Zishui, and Miluo Rivers of the Dongting Lake catchment, shows no shared haplotypes among these rivers and forms reciprocally monophyletic infra-lineages (Figure 2 and Figure 3). This finding supports our hypothesis that both Poyang and Dongting Lakes act as biogeographic barriers to gene flow among their respective tributary populations of rheophilic fishes. Previous studies have also demonstrated that the transition from lotic to lentic conditions in reservoirs can function as a biological filter, significantly restricting the downstream dispersal of rheophilic fishes [64,101,102]. Recent research has identified phylogeographic breaks among populations of two rheophilic fish species in the Poyang Lake tributaries [18,28]. In contrast, the eurytopic fish *Hemiculterella wui* (Cypriniformes: Xenocyprididae) in the same tributaries exhibits no clear geographic structure [103]. These results suggest that the “lake-barrier” effect on gene flow is likely mediated by species-specific life-history or behavioral traits.

K-IIb and K-IIc, two sister sub-lineages of *A. kreyenbergii*, are segregated between the Poyang and Dongting Lake catchments of the Yangtze basin. Ancestral-area reconstruction indicates that K-IIc originated in the Poyang system (node 5 in Figure 2) and later dispersed into the Dongting Lake catchment. The split, dated to ~0.8 Ma, coincides with a glacial interval characterized by cold–arid climates and sea-levels ~100 m lower than today [104,105]. Such conditions likely intensified headwater erosion and may have facilitated river-capture events across the low drainage divide of the Mufu, Lianyun, and Luoxiao mountain ranges. We therefore infer that K-IIc colonized the Dongting system via a capture-mediated transfer from the Poyang headwaters, and the intervening mountains have since acted as a topographic barrier fostering detectable genetic divergence between rheophilic fish populations of the two sub-catchments. Comparative phylogeographic surveys of additional fish and terrestrial taxa are required to evaluate the general role of these mountain ranges in driving intraspecific divergence across South–Central China.

Sub-lineage K-IIc is found concurrently in the Xijiang River (Pearl basin) and the Dongting Lake catchment (Yangtze River basin). Phylogenetic inference and ancestral-area reconstruction revealed that each haplotype in the Xijiang River is nested within the haplotype set of the Xiang River (the Dongting Lake tributary), pinpointing dispersal from the Xiang River into the Xijiang River at nodes 10 and 11 in Figure 2. The two rivers share three haplotypes, K1, K12, and K13 (Figure 3), a pattern most plausibly explained by dispersal through the Lingqu Canal. Built more than 2200 years ago in the Qin Dynasty [73], this artificial channel links the Xiang (Yangtze) directly with the Guijiang River (a tributary of the Xijiang River), allowing water to flow from the Yangtze into the Pearl drainage [74]. The haplotype footprint therefore supports the hypothesis that the canal has served as a fish dispersal corridor, enabling headwater stream fish to colonize upstream reaches of the Xiang and Xijiang Rivers in either direction. Earlier work [67,68,69,70] has already invoked the same historic waterway to explain the anomalously tight genetic affinity of fish populations now separated by the Nanling divide between the Xiang and Xijiang catchments.

### 4.2. Genetic Diversity and Demographic History

*A. kreyenbergii* exhibits high overall haplotype diversity (*h* = 0.795) and nucleotide diversity (*π* = 0.0135), fitting Grant and Bowen’s Type IV category (*h* ≥ 0.5, *π* ≥ 0.005) [106]. This high–high pattern represents the prevailing genetic signature of overall genetic diversity reported in Chinese freshwater fishes [6,12,13,15,18,28,29,31,33,35]. At the drainage scale, *A. kreyenbergii* in the Yangtze River exhibits the “high *h*, high *π*” profile produced by the co-occurrence of lineages K-I and K-II. In contrast, the Pearl and Huai Rivers each host a single lineage (K-I or K-II) and display “high *h*, low *π*” signals, the classic imprint of a historical bottleneck followed by rapid demographic expansion [106].

The fact that N_ST_ is obviously higher than G_ST_ indicates that closely related haplotypes tend to be geographically clustered. This points to a marked genetic differentiation among *A. kreyenbergii* populations, a pattern strongly corroborated by the high levels of genetic divergence revealed in pairwise comparisons (Table 2). Recent work on small rheophilic stream fishes across South–Central China [18,28,107,108] reveals a pronounced spatial genetic structure, indicating that small mid- to bottom-dwelling freshwater fishes are weak dispersers, prone to accumulating genetic divergence and, consequently, marked population differentiation [15].

Neutrality tests and mismatch-distribution analyses jointly indicate a recent rapid demographic expansion in the *A. kreyenbergii* sub-lineages K-IIa and K-IIc, with Bayesian skyline plots dating the onset of growth to ~0.014 and 0.0097 Ma, respectively. The rapid demographic growth detected in sub-lineages K-IIa (~0.014 Ma) and K-IIc (~0.0097 Ma) coincides closely with the post-Last Glacial Maximum (LGM, 0.026–0.019 Ma) strengthening of the East Asian summer monsoon, which has sustained warm humid conditions across the region since ~0.015 Ma [104,109,110,111]. Therefore, we propose that glacial cooling and aridification during the Late Pleistocene caused the effective population size in both sub-lineages to contract, and the subsequent rise in temperature and precipitation after the LGM released this constraint, triggering the pronounced and rapid population recoveries recorded in the genetic data. Post-LGM population expansions mirroring those that we document here have been repeatedly inferred for other Chinese freshwater fishes [6,112,113,114].

### 4.3. Conservation Considerations

Genetic diversity is the cornerstone of nature’s resilience, supporting population viability, adaptive potential, species persistence, and ecosystem resilience [115,116]. It enables populations to adapt to environmental changes, recover from disturbances, and ultimately sustain species and ecosystem diversity in the face of pressures [117,118]. In the context of accelerating global change, conservation efforts must now integrate the range-wide protection of genetic diversity and evolutionary potential with urgent genetics-based interventions [119,120,121]. This is essential to curb the population and genetic erosion that is sweeping through terrestrial, freshwater, and marine systems [122,123].

Our phylogenetic and SAMOVA analyses have revealed two major lineages and five sub-lineages in *A. kreyenbergii*, as well as four distinct genetic units. These lineages, sub-lineages, and genetic units should be recognized as Evolutionary Significant Units (ESUs), Conservation Units, or Management Units, in accordance with the criteria established by Moritz [124], Avise [125], and Coates et al. [126]. Our results support designating four genetically distinct conservation zones for *A. kreyenbergii*: the Pearl River and Dongting Lake catchments (combined), the Poyang Lake catchment, the Qiupu River, and the Huai River. Notably, three of these ESUs are found within the Yangtze–Poyang Lake catchment (Figure 1 and Figure 2), a region characterized by high genetic diversity (Table 1). Given this, we emphasize the Yangtze–Poyang Lake catchment as a priority area for conservation efforts aimed at preserving the genetic diversity of this species. This recommendation is further supported by a recent study highlighting the importance of this region for spatial conservation prioritization, specifically for the protection of the genetic diversity of the stream fish *S. parvus* (Cypriniformes: Gobionidae) [18]. Additionally, ecosystem size and spatial complexity are key drivers of freshwater biodiversity in natural river systems [127,128]. Therefore, effective conservation strategies for Chinese freshwater fishes must consider the spatial complexity of large river systems to protect their genetic diversity, as recommended by Li et al. [18].

## 5. Conclusions

To summarize, *A. kreyenbergii* comprises two main lineages and five sub-lineages, characterized by a pronounced phylogeographic structure. The divergence of these lineages and sub-lineages was primarily driven by geographic isolation stemming from multiple factors. The Late-Cenozoic collapse of the Zhe–Min Uplift and the Mufu–Lianyun–Luoxiao mountain chain as biogeographic barriers have significantly impeded gene flow between populations. Furthermore, the large Poyang and Dongting Lakes function as biological filters for rheophilic fishes, exacerbating genetic isolation. Additionally, the construction of the Lingqu Canal during the Qin Dynasty historically served as a dispersal corridor, facilitating population exchange between the Yangtze and Pearl Rivers, thereby influencing the genetic structure of the species. Given the spatial subdivision of the genetic structure of *A. kreyenbergii*, we recommend that the four conservation geographic units—namely, the Pearl River and Dongting Lake catchment, the Poyang Lake catchment, the Qiupu River, and the Huai River—be classified and managed as distinct entities. Within the Yangtze–Poyang Lake catchment, three provisional ESUs have been identified, and high overall genetic diversity has been observed. These findings underscore the importance of this region as a key spatial conservation priority area. Our research offers valuable insights for the development of management and conservation strategies aimed at preserving the genetic diversity of freshwater fish species in the coastal rivers of South–Central China.

## Figures and Tables

**Table 1:** Mitochondrial *Cyt b* genetic diversity in *A. kreyenbergii*.

River Basin or Catchment	No. of Individuals	No. of Haplotypes	No. of Private Haplotypes	Haplotype Diversity	Nucleotide Diversity
Pearl River	93	15	12	0.383 ± 0.065	0.0004 ± 0.0004
Huai River	7	2	2	0.286 ± 0.196	0.0005 ± 0.0005
Yangtze River	124	48	45	0.927 ± 0.014	0.0160 ± 0.0079
Dongting Lake catchment	57	19	16	0.776 ± 0.054	0.0029 ± 0.0017
Poyang Lake catchment	58	26	26	0.886 ± 0.030	0.0159 ± 0.0080
Qiupu River	9	3	3	0.639 ± 0.126	0.0007 ± 0.0006
Overall	224	62	59	0.795 ± 0.028	0.0135 ± 0.0067

**Table 2:** Pairwise Φ_ST_ matrix (lower left) and associated *p*-values (upper right) for *A. kreyenbergii*.

		Yangtze River			
	Pearl River	DLC	PLC	Qiupu River	Huai River
Pearl River		0.0000	0.0000	0.0000	0.0000
Dongting Lake catchment (DLC)	0.1182		0.0000	0.0000	0.0000
Poyang Lake catchment (PLC)	0.6990	0.5774		0.0000	0.0000
Qiupu River	0.9737	0.8456	0.3931		0.0000
Huai River	0.9911	0.9415	0.6890	0.9872	

## Data Availability

The newly generated sequences in this study are available on GenBank (http://www.ncbi.nlm.nih.gov (accessed on 13 August 2025)), under accession numbers: PV699891–PV699950, PX094884–PX094888.

## Supplementary Materials

The following supporting information can be downloaded at https://www.mdpi.com/article/10.3390/genes16121393/s1. Table S1: Sampling-site information and mitochondrial *Cyt b* haplotype distribution for *A. kreyenbergii*; Table S2: The collection number for *A. kreyenbergii* deposited in the Zoological Museum of Fudan University; Table S3: Model comparison for ancestral-area reconstruction of *A. kreyenbergii* using BioGeoBEARS.
