# Supplementary material for: Mountains, Lakes, and Ancient Drainage Networks Sculpt the Phylogeographic Architecture of the Stream Headwater Fish Acrossocheilus kreyenbergii in China
Source: Genes (Basel). 2025 Nov 21;16(12):1393. doi: 10.3390/genes16121393 (PMC12732399; doi:10.3390/genes16121393)
Supplement: Supplementary file 1 [file genes-16-01393-s001.zip › genes-3999524-supplementary.pdf]

SUPPLEMENTARY MATERIALS

Mountains, Lakes, and Ancient Drainage Networks Sculpt the Phylogeographic Architecture of the Stream Headwater Fish *Acrossocheilus kreyenbergii* in China

Yun Chen, Guangmin Deng, Ziyu Le and Cuizhang Fu *

State Key Laboratory of Wetland Conservation and Restoration, National Observations and Research Station for Wetland Ecosystems of the Yangtze Estuary, Ministry of Education Key Laboratory for Biodiversity Science and Ecological Engineering and Institute of Eco-Chongming, School of Life Sciences, Fudan University,
Shanghai 200438, China; 20110700132@fudan.edu.cn (Y.C.); 21110700097@m.fudan.edu.cn (G.D.); 23210700105@m.fudan.edu.cn (Z.L.)
* Correspondence: czfu@fudan.edu.cn

Table S1. Sampling-site information and mitochondrial *Cyt b* haplotype distribution for *A. kreyenbergii*.

Sampling locality	Coordinate	N	Haplotype (N)
Pearl River Basin: Xijiang River			
1. Congjiang Country	25.73°N, 108.98°E	2	K1(1), K2(1)
2. Sanjiang Country	25.78°N, 109.61°E	7	K1(6), K3(1)
3. Longsheng Country	25.81°N, 110.01°E	9	K1(7), K3(1), K4(1)
4. Liuzhou City	25.06°N, 109.29°E	8	K1(3), K5(5)
5. Hechi City	24.51°N, 108.67°E	9	K1(8), K6(1)
6. Yongfu Country	24.98°N, 109.99°E	10	K1(10)
7. Pingle Country	24.64°N, 110.65°E	7	K1(6), K7(1)
8. Yangshuo Country	24.78°N, 110.50°E	17	K1(15), K8(1), K9(1)
9. Guilin City	25.26°N, 110.30°E	13	K1(11), K10(1), K11(1)
10. Xingan Country	25.57°N, 110.48°E	11	K1(6), K8(1), K12(1), K13(1), K14(1), K15(1)
Yangtze River Basin: Zishui River, Dongting Lake catchment			
11. Shaoyang Country	27.22°N, 111.50°E	2	K16(1), K17(1)
Yangtze River Basin: Xiang River, Dongting Lake catchment			
12. Yiyang Country	26.57°N, 111.85°E	3	K1(2), K12(1)
13. Quanzhou Country	25.93°N, 111.09°E	8	K1(6), K18(1), K19(1)
14. Shuangpai Country	25.96°N, 111.67°E	4	K1(4)
15. Dao Country	25.53°N, 111.56°E	4	K1(3), K20(1)
16. Jiangyong Country	25.29°N, 111.33°E	4	K1(3), K13(1)
17. Jianghua Country	25.20°N, 111.61°E	6	K1(5), K21(1)
18. Lanshan Country	25.38°N, 112.21°E	3	K22(2), K23(1)
19. Jiahe Country	25.59°N, 112.39°E	3	K1(1), K22(2)
20. Zixing City	25.95°N, 113.27°E	4	K24(4)
21. Rucheng Country	25.67°N, 113.70°E	4	K24(2), K25(2)
22. Anren Country	26.71°N, 113.24°E	3	K1(1), K26(1), K27(1)
23. You Country	26.99°N, 113.33°E	2	K28(1), K29(1)
24. Chaling Country	26.79°N, 113.54°E	1	K22(1)
25. Yanling Country	26.49°N, 113.77°E	4	K1(1), K22(1), K28(1), K30(1)
Yangtze River Basin: Miluo River, Dongting Lake catchment			
26. Pingjiang Country	28.66°N, 113.65°E	2	K31(2)
Yangtze River Basin: Xiu River, Poyang Lake catchment			
27. Tonggu Country	28.44°N, 114.19°E	4	K32(1), K33(1), K34(1), K35(1)
28. Xiushui Country	29.02°N, 114.54°E	8	K35(1), K36(2), K37(1), K38(1), K39(1), K40(1), K41(1)
29. Jingan Country	28.85°N, 115.39°E	14	K42(13), K43(1)
30. Anyi Country	28.84°N, 115.56°E	2	K42(2)
Yangtze River Basin: Xin River, Poyang Lake catchment			
31. Guixi Country	28.03°N, 117.10°E	3	K44(1), K45(1), K46(1)
32. Yanshan Country	28.32°N, 117.74°E	5	K47(4), K48(1)
33. Shangrao City	28.44°N, 118.18°E	19	K45(1), K47(9), K48(1), K49(1), K50(2), K51(2), K52(1), K53(1), K54(1)
Yangtze River Basin: Rao River, Poyang Lake catchment			
34. Wuyuan Country	29.37°N, 117.91°E	3	K55(1), K56(1), K57(1)
Yangtze River Basin: Qiupu River			
35. Shitai Country	30.21°N, 117.50°E	9	K58(1), K59(5), K60(3)
Huai River Basin			
36. Yingshan Country	31.12°N, 115.83°E	6	K61(6)
37. Luoshan Country*	31.83°N, 114.39°E	1	K62(1)

* The characteristics of this sample were originally documented by Zhou et al. [77].

Table S2. The collection number for *A. kreyenbergii* deposited in the Zoological Museum of Fudan University.

Sampling locality	N[a]	Collection number
1. Congjiang Country	2	AKCJ20205901, AKCJ20205902
2. Sanjiang Country	7	AKSJ201792301–AKSJ201792307
3. Longsheng Country	9	AKLS201792601–AKLS201792609
4. Liuzhou City	8	AKLZ202481201–AKLZ202481208
5. Hechi City	9	AKHC201792101–AKHC201792109
6. Yongfu Country	10	AKYF201771401–AKYF201771410
7. Pingle Country	7	AKPL201771601–AKPL201771607
8. Yangshuo Country	17	AKYS20147801–AKYS20147817
9. Guilin City	13	AKGL201771401–AKGL201771413
10. Xingan Country	11	AKXA20147901–AKXA20147911
11. Shaoyang Country	2	AKSY201471101, AKSY201471102
12. Yiyang Country	3	AKYY201772301–AKYY201772303
13. Quanzhou Country	8	AKQZ201771601–AKQZ201771608
14. Shuangpai Country	4	AKSP201873001–AKSP201873004
15. Dao Country	4	AKDX201872901–AKDX201872904
16. Jiangyong Country	4	AKJY201872901–AKJY201872904
17. Jianghua Country	6	AKJH201471001–AKJH201471006
18. Lanshan Country	3	AKLanS201872801–AKLanS201872803
19. Jiahe Country	3	AKJiaH201872701–AKJiaH201872703
20. Zixing City	4	AKZX201872301–AKZX201872304
21. Rucheng Country	4	AKRC201872101–AKRC201872104
22. Anren Country	3	AKAR201872601–AKAR201872603
23. You Country	2	AKYX201872701, AKYX201872702
24. Chaling Country	1	AKCL201872501
25. Yanling Country	4	AKYL201872501–AKYL201872504
26. Pingjiang Country	2	AKPJ2014121201, AKPJ2014121202
27. Tonggu Country	4	AKTG201241301–AKTG201241304
28. Xiushui Country	8	AKXS201241201–AKXS201241208
29. Jingan Country	14	AKJA201241501–AKJA201241514
30. Anyi Country	2	AKAY201772801, AKAY201772802
31. Guixi Country	3	AKGX201232901–AKGX201232903
32. Yanshan Country	5	AKYanS201771101–AKYanS201771105
33. Shangrao City	19	AKSR201771101–AKSR201771119
34. Wuyuan Country	3	AKWY201232701–AKWY201232703
35. Shitai Country	9	AKST201571501–AKST201571509
36. Yingshan Country	6	AKYingS202481501–AKYingS202481506
37. Luoshan Country[b]	1	

[a] The sample size from each sampling locality.

[b] This sample was originally sourced from Zhou et al. [77].

Table S3. Model comparison for ancestral-area reconstruction of *A. kreyenbergii* using BioGeoBEARS. The best-fitting model is indicated in bold.

**Model**	***ln L***	**Dispersal**	**Extinction**	**Founder event speciation**	**AICc**	**AICc_wt**
DEC	-66.820	0.130	0.029	0.000	137.800	0.007
**DEC+J**	**-60.890**	**0.064**	**0.000**	**0.016**	**128.200**	**0.840**
DIVALIKE	-72.560	0.190	0.000	0.000	149.300	0.000
DIVALIKE+J	-62.680	0.076	0.000	0.019	131.800	0.140
BAYAREALIKE	-78.920	0.063	1.320	0.000	162.100	0.000
BAYAREALIKE+J	-65.020	0.049	0.000	0.023	136.400	0.014
